# Brief motivational intervention for adolescents treated in emergency departments for acute alcohol intoxication – a randomized-controlled trial

**DOI:** 10.1186/1471-227X-14-13

**Published:** 2014-06-30

**Authors:** Silke Diestelkamp, Nicolas Arnaud, Peter-Michael Sack, Lutz Wartberg, Anne Daubmann, Rainer Thomasius

**Affiliations:** 1 German Center for Addiction Research in Childhood and Adolescence, University Medical Center Hamburg-Eppendorf, Hamburg, Germany; 2 Department of Medical Biometry and Epidemiology, University Medical Center Hamburg-Eppendorf, Hamburg, Germany

**Keywords:** Emergency department, Adolescents, Brief intervention, Alcohol intoxication, Randomized-controlled trial

## Abstract

**Background:**

Alcohol misuse among youth is a major public health concern and numbers of adolescents admitted to the emergency department for acute alcoholic intoxication in Germany are recently growing. The emergency setting offers an opportunity to reach at-risk alcohol consuming adolescents and provide brief interventions in a potential “teachable moment”. However, studies on brief interventions targeting adolescents in emergency care are scarce and little is known about their effectiveness when delivered immediately following hospitalization for acute alcohol intoxication. In this protocol we present the *HaLT-Hamburg* trial evaluating a brief motivational intervention for adolescents treated in the emergency department after an episode of acute alcoholic intoxication.

**Methods:**

The trial design is a parallel two-arm cluster randomized-controlled trial with follow-up assessment after 3 and 6 months. N = 312 participants aged 17 years and younger will be recruited Fridays to Sundays in 6 pediatric clinics over a period of 30 months. Intervention condition is a manual-based brief motivational intervention with a telephone booster after 6 weeks and a manual-guided intervention for caregivers which will be compared to treatment as usual. Primary outcomes are reduction in binge drinking episodes, quantity of alcohol use on a typical drinking day and alcohol-related problems. Secondary outcome is further treatment seeking. Linear mixed models adjusted for baseline differences will be conducted according to intention-to-treat (ITT) and completers (per-protocol) principles to examine intervention effects. We also examine quantitative and qualitative process data on feasibility, intervention delivery, implementation and receipt from intervention providers, receivers and regular emergency department staff.

**Discussion:**

The study has a number of strengths. First, a rigorous evaluation of *HaLT-Hamburg* is timely because variations of the *HaLT* project are widely used in Germany. Second, prior research has not targeted adolescents in the presumed teachable moment following acute alcohol intoxication. Third, we included a comprehensive process evaluation to raise external validity. Fourth, the study involved important stakeholders from the start to set up organizational structures for implementation and maintaining project impact.

**Trial registration:**

Current Controlled Trials ISRCTN31234060 (April 30^th^ 2012).

## Background

Alcohol misuse and particularly episodic heavy drinking is a significant public health concern across contemporary societies [1]. In 2011, 47% of European students aged 15 to 16 years experienced alcohol intoxication at least once in their lifetime and 17% did so during the last month [2]. In Germany, the number of adolescents admitted to in-patient treatment for acute alcoholic intoxication (AAI) is recently growing. During the last years the number has more than doubled to over 26.000 in 2011 [3], with high rates of repeated episodes of alcohol intoxication if not treated adequately [4].

Personal health risks associated with AAI for adolescents have been widely documented, including aggressive [5,6] and risky sexual behavior [7] and elevated mortality rates through injury [8] and traffic accidents [9]. Moreover, heavy episodic drinking in adolescence is associated with a number of social and developmental problems, such as deleterious effects on neurocognitive and hormonal development [10-12] and cognitive and emotional abilities [13-15]. Social conflicts, delinquency and problems of academic adjustment are often associated with repeated episodes of heavy drinking [16,17] also puts youth at risk for chronification of problematic substance use patterns into adulthood [18,19]. Beyond these immense personal risks, alcohol-related problems also impose significant economic burdens on public health care [20]. Thus, excessive alcohol use in adolescents continues to be a major public health problem [21,22] and indicated preventive interventions as early as in adolescence are essential [23,24].

### Current evidence for brief alcohol interventions

To date, brief interventions (BI’s) are among the most empirically supported individual level interventions for reducing alcohol use and alcohol-related problems in adolescents [25,26]. They are often based on principles of Motivational Interviewing (MI) [27], which is characterized by an empathic approach to the client and a non-judgemental and non-confrontative counseling style [28]. Such short-term preventive or therapeutic interventions usually span one to three sessions [29,30] with the goal to establish and support intrinsic motivation for behavior change and/or further treatment seeking [31]. A comprehensive body of evidence documents the usefulness of BI’s for problematic alcohol use in primary care [32] and general hospitals [29]. The emergency department (ED) has been identified as a feasible setting to implement early interventions for problematic alcohol use [33,34] and efficacy of BI’s in ED has attracted a substantial body of research [35-37]. However, with few exceptions [4,38-41] to date adolescents have been only sparsely addressed [42,43]. None the less a small number of studies indicate feasibility and effectiveness of BI’s for adolescents in an ED following an *alcohol-related event* such as alcohol-induced injury [38-40]. While these studies vary substantially in key conceptual and methodological issues and the heterogeneity of findings limits generalization [Diestelkamp SD, Drechsel M, Arnaud N, Thomasius R: *Brief Interventions for Alcohol-involved Adolescents in Emergency Care: A Systematic Review,* forthcoming] [43], these studies are informative because they support the notion that alcohol-related events causing hospitalization are associated with a “teachable moment” that opens a window of opportunity for effective intervention [44-46]. The experience of a potentially life-threatening AAI resulting in hospitalization supposedly leaves adolescents in a state of increased responsiveness to alcohol-related counseling [46,47]. While awareness of alcohol having prompted ED hospitalization generally influences BI outcomes [45], there is currently a lack of studies addressing the potentials of BI’s following AAI hospitalization. Given the articulated need for indicated preventive interventions for AAI we build on the current empirical and conceptual base for BI’s in this context as well as our own favourable pilot results for feasibility and initial effectiveness in this context [4]. In this study protocol we present the design and current implementation of a randomized-controlled trial that aims at evaluating effectiveness of a manualized brief motivational intervention (BMI) (indicated intervention) for children and adolescents who are being treated in the ED immediately following AAI, an approach that has been established in Germany and other European countries [48-50] over the last 10 years (see below) but has not been rigorously tested to date. Moreover, we include additional evaluative components to address practical conditions within the “real-world” ED-setting that might affect effectiveness and implementation [51,52].

### *HaLT-Hart am LimiT* (“Stop – close to the limit”)

*HaLT*[53] is a German alcohol prevention project that involves a broad network of cooperating institutions to pursue the goal of early prevention of heavy alcohol use among children and adolescents [54]. It is one of the most broadly applied alcohol-specific prevention projects for under 18 year-olds and is currently implemented in more than 160 locations across Germany. It was initiated against the background of growing numbers of children and adolescents in need of emergency medical care following an episode of AAI and growing evidence that adolescents with at-risk alcohol consumption can best be reached in the ED setting [55]. *HaLT* involves two strategies. First, a *proactive* or structural prevention component which aims at promoting responsible alcohol use through outreach work in schools, informing festival organizers and pub owners about risks of underage drinking and providing support for correct implementation of alcohol-specific regulations for the protection of minors. Second, a behavior-oriented or *reactive* component which includes an individual level “bridging session” targeting children and adolescents in the ED following an AAI and a brief consultation for their caregivers. The reactive component is topic to the study presented in this protocol. The “bridging session” is a single-session, semi-structured indicated preventive intervention based on core principles of MI and implemented by trained facilitators before discharge from hospital. Facilitators provide information on risks associated with excessive drinking and strategies for reducing these risks while highlighting personal responsibility for behavior change. They aim at raising awareness for consequences of risky alcohol consumption and establishing a positive relationship in order to motivate adolescents to take part in an experience-oriented group-training (*risk-check*) for risk-related competences which is offered by a cooperating counseling agency as part of the *HaLT* project (not further addressed in this protocol). Parents or caregivers are also adressed in hospital in order to enhance their motivation to support their child in participating in the group-training. To date, *HaLT* services have been partly evaluated but not using rigorous evaluation methods (RCT). Moreover, existing results [54,56-58] are difficult to generalize because in the field *HaLT* is practically implemented in a broad spectrum of modulations and lacks standardized procedures in intervention content and delivery. The *HaLT-Hamburg* intervention, which is subject of the trial presented in this protocol, includes a number of further developments when compared with *HaLT*. First, a theory-based manualized BMI including a counseling session with caregivers. Second, a manualized training for facilitators in delivering the *HaLT-Hamburg* BMI. Third, definition of standards regarding qualification of facilitators. Fourth, regular clinical group supervision for facilitators and fifth, a pragmatic manual adherence monitoring. Internationally, to our knowledge we are the first to evaluate effectiveness of a BMI for adolescents admitted for AAI in the ED using a randomized-controlled design.

### Objectives and hypotheses

The objective of this study is to evaluate the effectiveness of a manual-based BMI for adolescents admitted for AAI in the ED. Effectiveness will be evaluated by expected reductions of binge-drinking frequency (5 or more standard drinks at one occasion (4 for female) [59]), quantity of alcohol consumption on a typical drinking day and alcohol related problems at 3 and 6 month follow-up. Our primary hypothesis is that children and adolescents under the age of 18 years who receive the manualized BMI following AAI hospitalization will show lower levels on these outcomes when compared to controls not receiveing this treatment. We also include further health care utilization in response to the BMI as secondary outcome. Help seeking in the care system as recommended in our BMI is closely related to intention to change [60] and appropriate for additionally judging intervention effectiveness. We thus hypothesize that children and adolescents who receive the BMI will significantly more often access further counseling regarding alcohol use in the 6 months following hospitalization than children and adolescents who do not receive the BMI. Moreover, we will examine a number of expected moderating variables such as psychopathological symptoms, drinking history, concurrent substance use and family environment. Finally, alongside our RCT we include additional evaluation components that focus on process, context and practical implications for BMI delivery under “routine conditions” in the ED setting.

## Methods

### Trial design, setting and time frame

**Figure 1:**
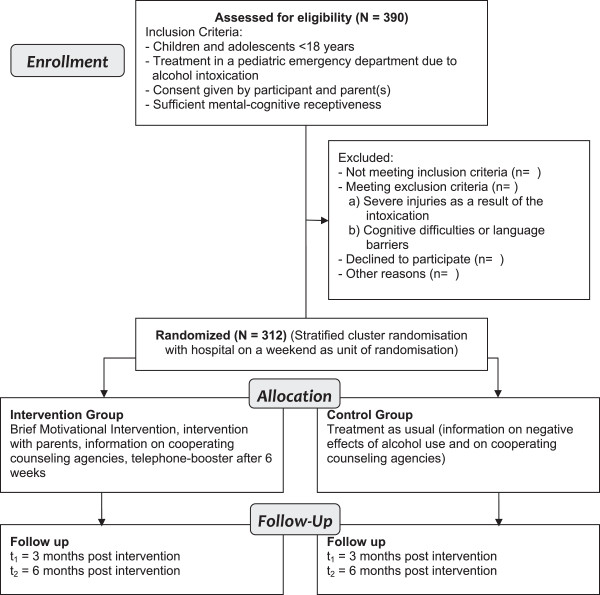
CONSORT flow diagram with anticipated case numbers.

The *HaLT-Hamburg* study is a parallel two-arm (intervention and control) stratified cluster RCT with follow-up assessments at 3 and 6 months post intervention with hospital on a weekend as unit of randomization and weekend as stratum. All participants receive standard inpatient ED care for AAI. Participants in the intervention group additionally receive a single session manualized BMI before ED discharge with one telephone booster session 6 weeks after the BMI. Caregivers of adolescents in the intervention group also receive a short manual-guided intervention by the same facilitator that delivered the BMI to the adolescent. Participants in the control group receive treatment as usual (TAU) only which is written information on negative consequences of alcohol use in adolescence and information on youth specific substance use counseling agencies. Recruitment of hospitals started in February 2011. Participant data collection started in July 2011 and lasts 30 months with final follow-up assessments being planned for July 2014. Figure 1 displays the CONSORT flow diagram of the study design.

#### Participating hospitals

ED directors of six pediatric hospitals identified as main treatment providers for alcohol-intoxicated adolescents under the age of 18 [61] in the City of Hamburg, Germany, were invited to a network conference at project launch, informed about the aims and procedures of the project and asked to participate. The conference was hosted by the Hamburg authorities for Health and Consumer Safety (BGV) who cooperates closely in project implementation. It was made clear that BMI’s will be delivered by external trained facilitators funded by health insurers since high workload and limited resources by ED staff are widely perceived as barriers for implementation in this context [34]. All six clinics spanning the area of the city agreed to participate in the study. In order to reach as many eligibles as possible the BGV issued an instruction for the Hamburg rescue coordination center to transfer adolescents with AAI to the six participating ED’s.

### Participants

#### ***Eligibility***

Study participants are children and adolescents admitted for AAI (diagnosis F10.0; ICD 10; [62]) in one of the participating clinics and their caregivers. They are eligible for participation if they fulfill the following inclusion criteria: 1. at hospitalization they are under the age of 18 years, 2. at time of data collection and intervention delivery they have sufficiently recovered from AAI and show sufficient mental-cognitive receptiveness, 3. they are fluent in German, 4. informed consent is given by participant and parent(s)/caregiver(s), 5. absence of severe injuries. We have purposefully limited exclusion criteria and designed inclusion/exclusion criteria for study participation to reflect actual clinical conditions. In principle, all individuals that would receive the BMI under “real” clinical conditions are eligible to participate in the study. This way we aim at maximizing external validity of our study findings. The age limit reflects the age range adressed in the German-wide *HaLT* project.

#### ***Recruitment and procedure***

Given that previous studies indicate that in Germany most AAI’s in youth happen at Friday and Saturday night [61], and an everyday stand-by recruitment was not possible due to limited resources, we recruit participants in the participating clinics on Friday, Saturday and Sunday mornings (between 7 – 9 am). Recruitment and data collection is carried out by trained research assistants and intervention delivery by trained facilitators who form a mobile intervention team. Coordination of attendance and resource availability is managed by a detailed operation schedule for each weekend. At each weekend in the evaluation period, research assistants contact all participating hospitals and ask about under 18 year-olds admitted to ED following AAI. If this is the case and if the patient has not been discharged in the same night, a research assistant visits the patient in hospital, informs about the project (evaluation and intervention), confidentiality, voluntariness of participation and right to withdraw consent and obtains active informed consent to participate in the study from patients and caregivers. Patients in the intervention and the control group are informed that they are compensated with incentives (shopping vouchers) summing up to €60 for complete data collection (€10 for baseline assessment and €25 for each completed follow-up assessment). If eligible patients and their caregivers who are willing to give consent are hospitalized in a clinic that was randomly assigned to form the intervention group for this weekend, the research assistant contacts one of the facilitators to deliver the BMI. Adolescents in the intervention group are contacted by telephone 6 weeks after hospitalization for a 5–10 minute manualized booster session to enhance motivation to pursue alcohol-related goals as set in hospital. All study participants are assessed via telephone for follow-up at 3 and 6 months post intervention. Approval for the study was obtained from the ethics committee of the Chamber of Psychotherapists Hamburg (Germany) prior to data collection. The study is conducted in accordance with CONSORT guidelines (Additional file 1) and is registered under Current Controlled Trials ISRCTN31234060 (http://www.controlled-trials.com/ISRCTN31234060).

### BMI condition

#### ***HaLT-Hamburg brief intervention***

The BMI is based on the German prevention program “*HaLT-Hart am Limit*” (“*Stop – close to the limit*”) and was adapted in a participatory process with cooperating practice partners, a youth-specific substance use counseling agency and an outpatient clinic for adolescents and young adults with substance use disorders. It is based on MI [28] and components reflect BMI elements as put forward by Spirito et al. [38]. The intervention is manual-based and consists of one 45-minute session. It’s 5 components are: 1. Introduction to the session with positive feedback on patient’s willingness to engage in the intervention, expression of interest and concern transporting a positive and empathic therapeutic mindset and explanation of the intervention’s aim and content. 2. A semi-structured interview assessing circumstances of the intoxication and alcohol-related risk behaviors. 3. Exploration phase incorporating discussion of motivation to drink, normative feedback, exploring pros and cons of current alcohol use, optional use of MI tools (i.e., importance and confidence ruler, decisional balance sheet) and establishment of future scenarios with changed/unchanged alcohol use. 4. Summary in which the facilitator structures and sums up what has been discussed, highlights personal responsibility for change and asks the patient for his/her conclusion from what has been discussed so far. 5. Closure of the session beginning with identification of drinking goals and potential barriers and development of strategies for goal attainment. The session is finished with a written agreement on drinking goals, the introduction of the cooperating youth-specific counseling agency and promotion of patient’s self-efficacy.

#### ***Counseling session for caregivers***

Parents maintain significant influence on adolescent’s alcohol use and parental integration has been proposed to greatly enhance efficacy of targeted prevention programs [63]. When caregivers pick up the adolescent in hospital they can easily be reached and are offered a brief consultation by the same facilitator who delivered the BMI to the adolescent. Caregivers are encouraged to reflect on the AAI episode of the minor and develop strategies to prevent future risky alcohol use. They are provided with general information on alcohol and alcohol-related risks and are encouraged to seek further family- and/or substance use related services if required. Afterwards parents, facilitator and adolescent get together for a summary statement that focuses on supporting the adolescent’s sense of self-efficacy with regards to the attainment of his/her alcohol-related goals.

#### ***Telephone booster***

Adolescents are contacted by telephone 6 weeks after discharge from hospital. The booster session is structured (manual-guided), lasts about 5–10 minutes and aims at enhancing content of the BMI and increasing motivation to pursue alcohol-related goals as set in hospital.

### Control condition

Participants in the control group are approached by a research assistant. After informed consent is given, they receive TAU which presently consists of oral and written information on cooperating youth- and family-orientated counseling agencies combined with the recommendation to contact a counseling agency and a flash drive with information on negative consequences of alcohol misuse for children and adolescents. Personal contact for TAU has a duration of 5–10 minutes.

### Treatment fidelity

Generally, the intervention is designed in a standardized way, yet it leaves facilitators with a certain leeway. This mentality reflects practitioners needs for flexibility and empathic focus on the client’s concerns in a MI spirit while keeping structure and content of the intervention sufficiently standardized [64]. Treatment fidelity is maintained using several strategies. First, the intervention (as the counseling session with parents/caregivers) is manualized. It provides clear guidelines and steps to be followed when carrying through the BI. Guidance is further enhanced by a short “memo-card”, which includes cues for core elements of the introduction, interview, exploration, summary and closure to the session and a reminder of the optional use of MI-tools. Additionally we developed a guide for delivering the telephone-booster. Second, all facilitators have a masters degree in psychology, social education work or related fields and are experienced in working with minors and their parents. Third, facilitators are initially trained by experienced and certified trainers (a clinical psychologist, a social educational worker and a research psychologist) in MI skills and in delivering the manualized BMI (12 hrs of training). On a bi-monthly basis facilitators are clinically supervised to discuss problems and experiences of implementation, engange in role-plays and receive retraining if required. Moreover, the manual was developed in a participatory approach by a team of experienced professionals including social education workers and senior clinical psychologists to raise practitioners acceptance and secure practicability under clinical conditions.

### Measures

Primary outcomes of the trial are reductions of past-month binge-drinking frequency, past-month quantity of alcohol consumption on a typical drinking day and alcohol-related problems in the past 3 months. We define binge drinking as consumption of 5 (4 for girls) or more alcoholic drinks at one occasion [59] and consider it as adequate primary outcome because it increases the risk for and often precedes AAI [4]. To assess binge drinking frequency we use a single question that is adapted from the Alcohol Use Disorder Identification Test Consumption subscale (AUDIT-C) [65] as used in a previous study [66]. Additional primary drinking outcome will be quantity of alcohol consumed on a typical drinking day as another indicator of risk for repeated AAI. For both alcohol measures (binge drinking and quantity of alcohol intake on a typical drinking day), we consider one unit of alcohol (standard drink) to include 10 g ethanol and we use a graphical overview of various types of drinks to help respondents answer the question and to ensure standardized responses. To assess alcohol-related behavioral problems we use the Rutgers Alcohol Problems Index (RAPI) [67] which is widely used and valid for an adolescent target population [68-71]. The secondary outcome concerns further seeking of counseling for alcohol use, which is retrospectively assessed by a single dichotomous (yes/no) question at both follow-ups and details on access and duration of services used.

Additionally we assess concurrent substance use following the assessment standards III of the German Society for Addicition Research and Therapy [72], repeated hospitalization due to AAI, general psychopathology through a short version of the Symptom Checklist SCL-9-K [73] and the Screening of Psychological Disorders in Adolescence (SPS-J) [74] as a behavioral screening instrument for early detection of externalizing and internalizing problems. Furthermore we assess readiness to change through an algorithm [75] allowing allocation of individuals to the different stages of change as proposed by Prochaska and DiClemente [76] and alcohol-related cognitive variables such as knowledge (modified from [77], social norms [77], self-efficacy (selected items of the Alcohol Abstinence Self Efficacy Scale (AASE-G) [78]) and attitudes through a 9-item semantic differential [79]. All assessment instruments are based on self-reports. We collect basic demographic data on age, gender, ethnic- and socioeconomic family status.

### Additional components of evaluation

As mentioned above we include additional components to evaluate effectiveness alongside our RCT design. In summary, we examine quantitative and qualitative process data on intervention delivery, implementation and receipt from intervention providers, receivers and regular ED staff which is guided by the framework of RE-AIM (Reach, Effectiveness, Adoption, Implementation, and Maintenance) as suggested by Glasgow and colleagues [80]. This framework represents a systematic approach to the evaluation of research translation potentials into practice and has been applied for the evaluation of implementation of BI’s in ED before [34].

#### ***Intervention implementation and delivery***

We will explore qualitative contextual information about AAI treatment in the participating clinics and whether the *HaLT-Hamburg* intervention is familiar to the clinic staff. Furthermore we examine attitudes, level of interest/commitment and perceived barriers for routine implementation among ED staff (medicals, nurses) and BMI facilitators. This information includes structural conditions affecting delivery (e.g., is the BMI delivered in a seperate room or a corridor with hospital staff, patients and visitors passing by) as well as duration of session and type and duration of possible interruptions. Facilitators complete a short record indicating details about intervention delivery (such as content, MI-techniques used, referral to further counseling) after each BI. Finally, heads of departments will be interrogated providing information on perceived barriers and ressources for long term project implementation.

#### ***Receipt and acceptance of BMI***

Patient’s acceptance of the intervention is evaluated with the Treatment Satisfaction Questionnaire (Fragebogen zur Patientenzufriedenheit ZUF-8, [81]) using 8 items (e.g. “To what extend did the counselling session meet your needs?”). For adherence to MI spirit patients rate their perception of facilitator’s therapeutic skills with 8 items (e.g. “the facilitator respects me”, “the facilitator seems empathic”) on a 4-point response scale immediately after the BI session in hospital (Index of Basic Therapeutic Skills (BIS) (short version) [82].

#### ***Feasibility***

Similar to the approach of Linakis et al. [23] we assess feasibility through the number of enrolled participants and the number of those who complete all elements of the intervention. An indication for feasibility will be given if at least 80% eligibles participate and 90% or more of those who participate complete the intervention before discharge from hospital.

### Randomization

Because of limited resource availability, individual level random assignment of eligible participants to BMI or control condition is not feasible in this study. Instead a stratified cluster randomization is deemed appropriate with weekends (N = 129) as stratum and hospital (N = 6) on a weekend as unit of randomization. Over a data collection period of 30 months this yields a total of N = 774 possible clusters. This approach leads to a high number of clusters, which is highly recommended [83] and assures that at each weekend patients in one half of the hospitals are assigned to the BMI and the other half to the control condition. The total amount of possible combinations between strata and hospitals is balanced in a way that each clinic acts equally often as control and BMI condition and assures that all clinics are either control or BMI condition at each weekend. The resulting randomization plan was established prior to the data collection process by a research assistant from the Department of Medical Biometry and Epidemiology (University Hospital Hamburg-Eppendorf) who is not involved in the project, using the statistical software package SAS, Version 9.3 [84].

### Sample size

The sample size is calculated for the three primary outcomes binge-drinking frequency, quantity of alcohol consumption on a typical drinking day and alcohol related problems at 3 month follow-up. Type I error is set to 5% for each of these outcomes. With an effect size of 0.26 and a power of 80%, 2 × 153 patients need to be included if randomization occurs at patient level. With an assumed intra-cluster correlation of 0.05 and an average of 1.264 included patients per cluster, we calculated a design effect of 1.013. Hence, the required sample size increases to 156 patients and 154 clusters per group, resulting in a total sample of N = 312. Clusters (hospitals per weekend) without patients will not be included in the analysis. With an expected participation rate of 80%, we anticipate N = 390 ED patients to be assessed for eligibility over a recruitment period of 30 months in the six participating hospitals. Based on a prior pilot-study [4], this sample size is feasible.

### Statistical analysis

Basic descriptive statistics will be calculated for baseline variables, and according to trial arm (BMI vs. control). Intention-to-treat (ITT) analysis will be based on available data from all randomized patients at 3 and 6 month follow-up. In case of missing follow-up values multiple imputation will be performed [85]. For primary and secondary hypotheses we will use linear mixed models adjusted for baseline differences to examine differences between intervention and control group with intervention condition as fixed effect and clusters (hospital on a weekend) as random effect. The two-sided ?-level is set to 0.05. Additional analysis will be conducted on a per-protocol analysis set. Process data on intervention delivery, implementation and receipt will be analyzed in subsequent steps.

## Discussion

In this study protocol we present the design and current implementation of a randomized-controlled trial which aims at evaluating the effectiveness of the indicated *HaLT-Hamburg* intervention for children and adolescents following treatment due to AAI in the ED. Beside a rigorous trial design with focus on relevant alcohol-related outcomes, we include additional evaluative components to address important issues of feasibility and practical implementation under “real-world” conditions in an ED-setting.

### Strengths and limitations

The study has a number of significant strengths. First and foremost, a rigorous evaluation of this intervention is timely because *HaLT* interventions are already widely applied in Germany in different modulations. *HaLT* interventions are brief and based on MI, which has been proven effectiv for reducing alcohol-related problems [64,86]. Previous study results are promising [54,56-58], yet difficult to generalize and full scale trial evaluations including process and implementation evaluation are missing to date. Overall, with our study we contribute to the literature mainly because studies addressing effectiveness of BI’s in the ED with a focus on minors hospitalized following an alcohol-related event are scarce [Diestelkamp SD, Drechsel M, Arnaud N, Thomasius R: *Brief Interventions for Alcohol-involved Adolescents in Emergency Care: A Systematic Review,* forthcoming] and most existing RCT’s in the ED setting have been conducted in the U.S., leaving uncertainty whether results generalize to other countries [87]. To our knowledge we are the first to target adolescents in the ED immediately after the experience of an AAI. This approach appears promising due to a presumed “teachable moment” arising from the potentially life-threatening experience of AAI leading to hospitalization. This experience provides a window of opportunity for initiating behavior change in minors who have elevated risks for repeated AAI episodes if untreated [4] and who are in a developmental stage in which risk behaviors are normative which may limit responsiveness to MI or brief advice in other settings [88].

Another strength is the integration of additional evaluative components via a comprehensive process evaluation which qualifies *HaLT-Hamburg* as a pragmatic trial [89,90] and raises external validity. In the considerations guiding our study we aimed at minimally interfering with “real world” conditions, as indicated by applying rather unrestricted inclusion (access-to-care) criteria, unobtrusive measures for intervention fidelity monitoring, employing facilitators who will continue working in the project after data collection has ceased (as opposed to research assistants) and a number of other aspects associated with implementation and intervention delivery. For example, while intervention, telephone booster and training sessions are manual-based and replicable, facilitators are left with a certain leeway in intervention delivery to allow tailoring of the intervention to the patient’s needs in order to adhere to MI spirit [64]. This may pose a limiting factor for internal validity. However, we included measures for manual adherence and MI-fidelity as well as clients’ ratings of perceived facilitator’s MI skills. A second possible limiting factor is that our data are based on self-reports. However, this approach is widely used in comparable studies [24] and previous studies indicate that adolescent self-reports on substance use are reasonably valid [91,92].

### Implications for practice

As mentioned above, our study addresses a number of practical aspects. Importantly, actions for setting up organizational structures to implementation involving relevant stakeholders have been considered from the beginning of the project. This way, funding of ED external trained facilitators could be assured by partnering health insurances during the study period and with a clear perspective of further funding if our intervention proves effective. Moreover, our study is embedded in *psychenet: the Hamburg Network for Mental Health*[93] with over 60 partners from research, health care, health industry and government in the Free and Hanseatic City of Hamburg. This network provides a strong structural resource for further implementation. *HaLT-Hamburg* is thus well embedded and supported by communal structures, is implemented under “real world” clinical conditions and is therefore suited to reveal important information on possible barriers and resources for practical implementation [60,94] in addition to insights on effectiveness and conditions influencing effectiveness of the HaLT-Hamburg BMI.

## Conclusion

Our study addresses a highly relevant target group and contributes to the current literature on brief interventions by filling apparent gaps. The study will provide insights about effectiveness of the *HaLT-Hamburg* intervention and hence about a promising approach of targeted interventions for adolescents experiencing AAI. Moreover, we integrated the evaluation of practical implications and address important elements of translational research as well as actions needed to sustainably implement BI’s under practical conditions, an issue often neglected in prior research.

## Abbreviations

AAI: Acute alcohol intoxication; BI: Brief intervention; BMI: Brief motivational intervention; ED: Emergency department; MI: Motivational interviewing; TAU: Treatment as usual

## Competing interests

The authors declare that they have no competing interests.

## Authors’ contributions

SD and NA drafted the manuscript. PMS and RT obtained funding. SD, PMS and RT designed the intervention and developed the study methodology in close cooperation with AD. AD calculated the sample size and established the randomization plan. SD developed the training and intervention manual. NA and RT coordinate the study. LW and SD are responsible for carrying through its organizational processes. All authors read and approved the final manuscript.

## Pre-publication history

The pre-publication history for this paper can be accessed here:

http://www.biomedcentral.com/1471-227X/14/13/prepub

## Supplementary Material

Additional file 1 CONSORT checklist. Click here for file

## References

[B1] Conrod PJ Castellanos-Ryan N Mackie C Long-term effects of a personality-targeted intervention to reduce alcohol use in adolescents J Consult Clin Psychol 2011 79 3 296 306 21500886 10.1037/a0022997

[B2] Hibell B Guttormsson U Ahlström S Balakireva O Bjarnason T Kokkevi A Kraus L The 2011 ESPAD report: Substance use among students in 36 European countries 2012 Stockholm: The Swedish council for information on alcohol and other drugs (CAN)

[B3] Gesundheitsberichterstattung des Bundes Diagnosedaten der Krankenhäuser ab 2000 [http://www.gbe-bund.de]

[B4] Stolle M Sack P-M Broening S Baldus C Thomasius R Brief Intervention in Alcohol Intoxicated Adolescent-A follow-up study in an access-to-care sample J Alcohol Drug Depend 2013 1 106

[B5] Swahn MH Simon TR Hammig BJ Guerrero JL Alcohol consumption behaviors and risk for physical fighting and injuries among adolescent drinkers Addict Behav 2004 29 5 959 963 15219342 10.1016/j.addbeh.2004.02.043

[B6] MacDonald S Cherpitel CJ Borges G DeSouza A Giesbrecht N Stockwell T The criteria for causation of alcohol in violent injuries based on emergency room data from six countries Addict Behav 2005 30 1 103 113 15561452 10.1016/j.addbeh.2004.04.016

[B7] Champion HLO Foley KL DuRant RH Hensberry R Altman D Wolfson M Adolescent sexual victimization, use of alcohol and other substances, and other health risk behaviors J Adolesc Health 2004 35 4 321 328 15450546 10.1016/j.jadohealth.2003.09.023

[B8] Sindelar HA Barnett NP Spirito A Adolescent alcohol use and injury: A summary and critical review of the literature Minerva Pediatr 2004 56 3 291 309 15252378

[B9] Viner RM Taylor B Adult outcomes of binge drinking in adolescence: findings from a UK national birth cohort J Epidemiol Community Health 2007 61 10 902 907 17873228 10.1136/jech.2005.038117 PMC2652971

[B10] Townshend JM Duka T Binge Drinking, Cognitive Performance and Mood in a Population of Young Social Drinkers Alcohol Clin Exp Res 2005 29 3 317 325 15770105 10.1097/01.alc.0000156453.05028.f5

[B11] Heffernan T Clark R Bartholomew J Ling J Stephens S Does Binge Drinking in Teenagers affect their Everyday Prospective Memory? Drug Alcohol Depend 2010 109 73 78 20071106 10.1016/j.drugalcdep.2009.12.013

[B12] McQueeny T Schweinsburg BC Schweinsburg AD Jacobus J Baya S Frank LR Tapert SF Altered White Matter Integrity in Adolescent Binge Drinkers Alcohol Clin Exp Res 2009 33 7 1278 1285 19389185 10.1111/j.1530-0277.2009.00953.x PMC2825379

[B13] Spear L Adolescent brain and the college drinker: biological basis of propensity to use and misuse alcohol J Stud Alcohol 2002 14 71 81 10.15288/jsas.2002.s14.71 12022731

[B14] Tapert SF Granholm E Leedy NG Brown SA Substance use and withdrawal: neuropsychological functioning over 8 years in youth J Int Neuropsychol Soc 2002 8 873 883 12405538 10.1017/s1355617702870011

[B15] Pharo H Sim C Graham M Gross J Hayne H Risky Business: Executive Function, Personality, and reckless Behavior During Adolescence and Emerging Adulthood Behav Neurosci 2011 125 970 978 22004262 10.1037/a0025768

[B16] Miller JW Naimi TS Brewer RD Jones SE Binge drinking and associated health risk behaviours among high school students Pediatrics 2007 119 1 76 85 17200273 10.1542/peds.2006-1517

[B17] Battin-Pearson S Newcomb MD Abbott RD Hill KG Catalano RF Hawkins JD Predictors of early high school dropout: A test of five theories Br J Educ Psychol 2000 92 3 568 582

[B18] Van Der Vorst H Vermulst AA Meeus WH Dekovic M Engels RC Identification and prediction of drinking trajectories in early and mid-adolescence J Clin Child Adolesc Psychol 2009 38 3 329 341 19437294 10.1080/15374410902851648

[B19] Stolle M Sack PM Thomasius R Binge Drinking in Childhood and Adolescence Epidemiology, Consequences, and Interventions Dtsch Arztebl Int 2009 106 19 323 19547732 10.3238/arztebl.2009.0323 PMC2689602

[B20] Toumbourou JW Stockwell T Neighbors C Marlatt GA Sturge J Rehm J Interventions to reduce harm associated with adolescent substance use Lancet 2007 369 9570 1391 1401 17448826 10.1016/S0140-6736(07)60369-9

[B21] Dawson DA Goldstein RB Chou SP Ruan WJ Gran BFT Age at First Drink and the First Incidence of Adult-Onset DSM-IV Alcohol Use Disorders Alcohol Clin Exp Res 2008 32 12 2149 2160 18828796 10.1111/j.1530-0277.2008.00806.x PMC2760820

[B22] Grant BF Stinson FS Harford TC Age at onset of alcohol use and DSM-IV alcohol abuse and dependence: a 12-year follow-up J Subst Abuse 2001 13 4 493 504 11775078 10.1016/s0899-3289(01)00096-7

[B23] Linakis JG Bromberg J Baird J Nirenberg TD Chun TH Mello MJ Jackson KM Spirito A Feasibility and Acceptability of a Pediatric Emergency Department Alcohol Prevention Intervention for Young Adolescents Pediatr Emerg Care 2013 29 11 1180 1188 24168879 10.1097/PEC.0b013e3182a9f7da PMC4340665

[B24] Lammers J Goossens F Lokman S Monshouwer K Lemmers L Conrod P Wiers R Engels R Kleinjan M Evaluating a selective prevention programme for binge drinking among young adolescents: study protocol of a randomized controlled trial BMC Public Health 2011 11 126 21338506 10.1186/1471-2458-11-126 PMC3053243

[B25] Moyer A Finney JW Swearingen CE Vergun P Brief interventions for alcohol problems: a meta-analytic review of controlled investigations in treatment-seeking and non-treatment-seeking populations Addiction 2002 97 279 292 11964101 10.1046/j.1360-0443.2002.00018.x

[B26] Bertholet N Daeppen JB Wietlisbach V Fleming M Burnand B Reduction of alcohol consumption by brief alcohol intervention in primary care: systematic review and meta-analysis Arch Intern Med 2005 165 9 986 995 15883236 10.1001/archinte.165.9.986

[B27] Walters ST Neighbors C Feedback interventions for college alcohol misuse: What, why and for whom? Addict Behav 2005 30 1168 1182 15925126 10.1016/j.addbeh.2004.12.005 PMC2459313

[B28] Miller WR Rollnick S Motivational Interviewing: Preparing People for change 2002 2 New York: The Guilford Press

[B29] McQueen J Howe TE Allan L Mains D Hardy V Brief interventions for heavy alcohol users admitted to general hospital wards Cochrane Database Syst Rev 2011 http://dx.doi.org/10.1002/14651858 10.1002/14651858.CD005191.pub3 PMC10600352 21833953

[B30] Babor TF Avoiding the horrid and beastly sin of drunkenness: does dissuasion make a difference? J Consult Clin Psychol 1994 62 6 1127 1140 7860811 10.1037//0022-006x.62.6.1127

[B31] Spijkerman R Roek MAE Vermulst A Lemmers L Huiberts A Engels RCME Effectiveness of a web-based brief alcohol intervention and added value of normative feedback in reducing underage drinking: a randomized controlled trial J Med Internet Res 2012 12 5 e65 21169172 10.2196/jmir.1465 PMC3057308

[B32] Kaner EF Dickinson HO Beyer FR Campbell F Schlesinger C Heather N Saunders JB Burnand B Pienaar ED Effectiveness of brief alcohol interventions in primary care populations Cochrane Database Syst Rev 2007 http://dx.doi.org/10.1002/14651858 10.1002/14651858.CD004148.pub3 17443541

[B33] Rumpf HJ Early intervention in the medical care of problematic alcohol use: On the way to implementation Sucht 2009 55 6 326 327

[B34] Bernstein E Topp D Shaw E Girard C Pressman K Woolcock E Bernstein J A Preliminary Report of Knowledge Translation: Lessons From Taking Screening and Brief Intervention Techniques From the Research Setting Into Regional Systems of Care Acad Emerg Med 2009 16 11 1225 1233 20053242 10.1111/j.1553-2712.2009.00516.x

[B35] Nilsen P Baird J Mello MJ Nirenberg T Woolard R Bendtsen P Longabaugh R A systematic review of emergency care brief alcohol interventions for injury patients J Subst Abuse Treat 2008 35 184 201 18083321 10.1016/j.jsat.2007.09.008

[B36] Havard A Shakeshaft A Sanson-Fisher R Systematic review and meta-analyses of strategies targeting alcohol problems in emergency departments: interventions reduce alcohol-related injuries Addiction 2008 103 368 376 18190671 10.1111/j.1360-0443.2007.02072.x

[B37] D’Onofrio G Fiellin DA Pantalon MV Chawarski MC Owens PH Degutis LC Busch SH Bernstein SL O’Connor PG A brief intervention reduces hazardous and harmful drinking in emergency department patients Ann Emerg Med 2012 60 2 181 192 22459448 10.1016/j.annemergmed.2012.02.006 PMC3811141

[B38] Spirito A Monti PM Barnett NP Colby SM Sindelar H Rohsenow DJ Lewander W Myers M A randomized clinical trial of a brief motivational intervention for alcohol-positive adolescents treated in an emergency department J Pediatr 2004 145 3 396 402 15343198 10.1016/j.jpeds.2004.04.057

[B39] Monti PM Colby SM Barnett NP Spirito A Rohsenow DJ Myers M Woolard R Lewander W Brief intervention for harm reduction with alcohol-positive older adolescents in a hospital emergency department J Consult Clin Psychol 1999 67 6 989 994 10596521 10.1037//0022-006x.67.6.989

[B40] Monti PM Barnett NP Colby SM Gwaltney CJ Spirito A Rohsenow DJ Woolard R Motivational interviewing versus feedback only in emergency care for young adult problem drinking Addiction 2007 102 8 1234 1243 17565560 10.1111/j.1360-0443.2007.01878.x

[B41] Spirito A Sindelar-Manning H Colby SM Barnett NP Lewander W Rohsenow DJ Monti PM Individual and family motivational interventions for alcohol-positive adolescents treated in an emergency department: results of a randomized clinical trial Arch Pediatr Adolesc Med 2011 165 3 269 274 21383276 10.1001/archpediatrics.2010.296 PMC3690344

[B42] Yuma-Guerrero PJ Velasquez MM von Sternberg K Maxson T Garcia N Screening, brief intervention, and referral for alcohol use in adolescents: a systematic review Pediatrics 2012 130 1 115 122 22665407 10.1542/peds.2011-1589

[B43] Newton AS Gokiert R Mabood N Ata N Dong K Ali S Vandermeer B Tjosvold L Hartling L Wild TC Brief emergency department interventions for youth who use alcohol and other drugs: a systematic review Pediatr Emerg Care 2013 29 5 673 684 23640153 10.1097/PEC.0b013e31828ed325

[B44] Williams EC Palfai T Cheng DM Samet JH Bradley KA Koepsell TD Wickizer TM Heagerty PJ Saitz R Physical Health and Drinking Among Medical Inpatients With Unhealthy Alcohol Use: A Prospective Study Alcohol Clin Exp Res 2010 34 7 1257 1265 20477765 10.1111/j.1530-0277.2010.01203.x PMC2911969

[B45] Walton MA Goldstein AL Chermack ST McCammon RJ Cunningham R Barry KL Blow FC Brief alcohol intervention in the emergency department: moderators of effectiveness J Stud Alcohol Drugs 2008 69 4 550 560 18612571 10.15288/jsad.2008.69.550 PMC3646582

[B46] Barnett NP Monti PM Wood MD Wagner EF, Waldron HB Motivational interviewing for alcohol-involved adolescents in the emergency room Innovations in adolescent substance abuse interventions 2001 Amsterdam: Pergamon/Elsevier Science Inc 143 168

[B47] Roper L McGuire J Salmon P Booth PG Treatment-seeking for alcohol problems: The influence of mirroring events and windows of opportunity Addict Res Theory 2013 21 479 488

[B48] Lang S Kuttler H Projekt HaLT - Hart am LimiT. Fruehintervention und kommunal verankerte Strategie zur Verhinderung von riskantem Rauschtrinken bei Kindern und Jugendlichen [Project Stop - Close to the Limit. Early intervention and community based strategies for the prevention of binge drinking in children and adolescents] Sucht 2007 30 1 27 37

[B49] Caflisch M Uldry V At the crossover of adolescent and alcohol Rev Med Suisse 2013 9 374 406 409 23477224

[B50] Fandler E Scheer P Rödl S Müller W Alkoholmissbrauch und -abhängigkeit bei Kindern und Jugendlichen [Alcohol misuse and dependence in children and adolescents] Monatsschr Kinderheilk 2008 156 591 604

[B51] Daeppen JB A meta-analysis of brief alcohol interventions in emergency departments: Few answers, many questions - Commentary Addiction 2008 103 3 377 378

[B52] Pantalon MV Martino S Dziura J Li FY Owens PH Fiellin DA O’Connor PG D’Onofrio G Development of a scale to measure practitioner adherence to a brief intervention in the emergency department J Subst Abuse Treat 2012 43 4 382 388 23021098 10.1016/j.jsat.2012.08.011 PMC3661016

[B53] Kuttler H Lang S Halt sagen – Halt geben. Ein Präventionsprojekt für Jugendliche mit riskantem Alkoholkonsum [Say stop and give support: A prevention project for adolescents with risky alcohol drinking behavior] Prevention 2004 1 24 26

[B54] Prognos The national pilot project HaLT – scientific monitoring [http://www.prognos.com/fileadmin/pdf/publikationsdatenbank/HaLT Short Report.pdf]

[B55] Healey C Rahmana A Faizal M Kinderman P Underage drinking in the UK: Changing trends, impact and interventions. A rapid evidence synthesis Int J Drug Policy 2014 25 124 132 24095678 10.1016/j.drugpo.2013.07.008

[B56] Reis O Papke M Haessler F Ergebnisse eines Projektes zur kombinierten Prävention jugendlichen Rauschtrinkens [Evaluation of a project for the prevention of adolescent binge drinking] Sucht 2009 55 6 347 356

[B57] Müller S Pabst A Kronthalwer F Grübl A Kraus L Burdach S Tretter F Akute Alkoholvergiftung bei Jugendlichen – Erste Ergebnisse eines Münchner Pilotprojekts [Acute alcohol intoxication in adolescents: preliminary results of a pilot project in Munich] Dtsch Med Wochenschr 2009 134 21 1101 1105 19437373 10.1055/s-0029-1222574

[B58] Wurdak M Wolstein J Motivbasierte Intervention am Krankenbett im Rahmen des Projektes HaLT –Hart am Abschlussbericht an das Bundesministerium für Gesundheit [Motiv-based intervention in hospital as part of the project “Stop - Close to the Limit”. Final report to the Federal Ministry of Health] [http://www.drogenbeauftragte.de/fileadmin/dateien-dba/DrogenundSucht/Alkohol/Downloads/AbschlussberichtMotivbasierteInterventionFin.pdf]

[B59] Herring R Berridge V Thom B Binge drinking: an exploration of a confused concept J Epidemiol Community Health 2008 62 6 476 479 18477743 10.1136/jech.2006.056721

[B60] Kypri K Methodological Issues in Alcohol Screening and Brief Intervention Research Subst Abus 2007 28 3 31 42 18077301 10.1300/J465v28n03_04

[B61] Sack PM Stolle M Thomasius R Erfassung alkoholintoxikierter Kinder und Jugendlicher bis 21 Jahre in Hamburg mittels eines Postkartenmeldesystems (E-AK21). Abschlussbericht an die BGS der Freien und Hansestadt Hamburg [Survey on alcohol intoxicated children and youths up to the age of 21 in Hamburg using a postcard registration system (E-AK21). Final report to the Office of Health of the Free and Hanseatic City of Hamburg] 2009 Hamburg: DZSKJ

[B62] World Health Organization ICD-10: International statistical classification of diseases and related health problems (10th Rev. ed.) 2011 Geneva: World Health Organization

[B63] Abar CC Examining the relationship between parenting types and patterns of student alcohol-related behavior during the transition to college Psychol Addict Behav 2012 26 20 29 21842968 10.1037/a0025108 PMC3243795

[B64] Hettema J Steele J Miller RW Motivational interviewing Annu Rev Clin Psychol 2005 1 91 111 17716083 10.1146/annurev.clinpsy.1.102803.143833

[B65] Saunders JB Aasland OG Babor TF de la Fuente JR Grant M Development of the Alcohol Use Disorders Identification Test (AUDIT): WHO Collaborative Project on Early Detection of Persons with Harmful Alcohol Consumption–II Addiction 1993 88 6 791 804 8329970 10.1111/j.1360-0443.1993.tb02093.x

[B66] Arnaud N Bröning S Drechsel M Thomasius R Baldus C Web-based screening and brief intervention for poly-drug use among teenagers: study protocol of a multicentre two-arm randomized controlled trial BMC Public Health 2012 12 826 23013141 10.1186/1471-2458-12-826 PMC3524050

[B67] Earleywine M LaBrie JW Pedersen ER A brief Rutgers Alcohol Problem Index with less potential for bias Addict Behav 2008 33 9 1249 1253 18547738 10.1016/j.addbeh.2008.05.006 PMC2504506

[B68] White HR Labouvie EW Towards the assessment of adolescents problem drinking J Stud Alcohol 1989 50 1 30 37 2927120 10.15288/jsa.1989.50.30

[B69] van Woerden N Smulders FTY de Jong PJ Implicit and explicit alcohol related cognitions in heavy and light drinkers J Abnorm Psychol 2002 111 4 648 658 12428778 10.1037/0021-843X.111.4.648

[B70] Wiers RW van de Luitgaarden J van den Wildenberg E Smulders FTY Challenging implicit and explicit alcohol-related cognitions in young heavy drinkers Addiction 2005 100 6 806 819 15918811 10.1111/j.1360-0443.2005.01064.x

[B71] Wiers RW Alcohol and drug expectancies as anticipated changes in affect: negative reinforcement is not sedation Subst Use Misuse 2008 43 3–4 501 516 10.1080/10826080701203021 18365942

[B72] Deutsche Gesellschaft für Suchtforschung und Suchttherapie (Hrsg.) Standards für die Durchführung von Katamnesen bei Abhängigen [Standards in conducting catamnesis in people with addictions] 1985 Freiburg im Breisgau: Lambertus

[B73] Klaghofer R Brähler E Konstruktion und Teststatistische Prüfung einer Kurzform der SCL-90-R [construction and evaluation of the short version of SCL-90-R] Z Klin Psychol Psychiatr Psychother 2001 49 2 115 124

[B74] Hampel P Petermann F SPS-J - Screening psychischer Störungen im Jugendalter. Deutschsprachige Adaptation des Reynolds Adolescent Adjustment Screening InventoryTM (RAASITM) von William M. Reynolds [SPS-J - Screening of psychological disorders in adolescence. German adaptation of the Reynolds Adolescent Adjustment Screening InventoryTM (RAASITM) by William M. Reynolds] 2005 Goettingen: Hogrefe

[B75] Heidenreich T Hoyer J Stadien der Veränderung bei Substanzmissbrauch und -abhängigkeit: Eine methodenkritische Übersicht [Stages of change in addictive behaviors: A methodological overview] Sucht 2001 47 3 158 170

[B76] Prochaska JO DiClemente CC Stages and processes of self-change of smoking: toward an integrative model of change J Consult Clin Psychol 1983 51 3 390 395 6863699 10.1037//0022-006x.51.3.390

[B77] Scheier LM Botvin GJ Expectancies as mediators of the effects of social influences and alcohol knowledge on adolescent alcohol use: a prospective analysis Psychol Addict Behav 1997 11 1 48 64

[B78] Bott KE Rumpf HJ Bischof G Meyer C Hannöver W Hapke U John U Gloeckner-Rist A, Küfner FR, Küfner H Alkoholabstinenz-Selbstwirksamkeitsfragebogen: Deutsche Version der Alcohol Abstinence Self-Efficacy (AASE) Scale [Alcohol Abstinence Self-Efficacy-Scale, German Version] Elektronisches Handbuch zu Erhebungsinstrumenten im Suchtbereich (EHES) [Electronic Handbook on Assessment Instruments in the Addiction Field], Volume 3 2003 [http://www.gesis.org/unser-angebot/daten-erheben/zis-ehes/download-ehes/]

[B79] Himmelfarb S Eagly AH Chaiken S The measurement of attitudes Psychology of Attitudes 1993 Fort Worth: Harcourt Brace 23 88

[B80] Glasgow RE Vogt TM Boles SM Evaluating the public health impact of health promotion interventions: the RE-AIM Framework Am J Public Health 1999 89 9 1322 1327 10474547 10.2105/ajph.89.9.1322 PMC1508772

[B81] Schmidt J Lamprecht F Wittmann WW Zufriedenheit mit der stationären Versorgung. Entwicklung eines Fragebogens und erste Validitätsuntersuchungen [Satisfaction with inpatient treatment. Development of a questionnaire and preliminary validity checks] Psychother Med Psychol 2002 39 248 255 2762479

[B82] Stucki C Die Therapiebeziehung differentiell gestalten. Intuitive Reaktionen, Patientenwahrnehmung und Beziehungsverhalten von Therapeuten in der Psychotherapie [Creating differential therapeutic alliances. Intuitive reactions, patient perception and therapeutic alliance in psychotherapy] University of Bern 2004 [http://www.zb.unibe.ch/download/eldiss/04stucki_c.pdf]

[B83] Donner A Klar N Statistical considerations in the design and analysis of community intervention trials J Clin Epidemiol 1996 49 435 439 8621994 10.1016/0895-4356(95)00511-0

[B84] SAS Institute Inc What’s new in SAS 9.3 2012 Cary, NC: SAS Institute Inc

[B85] van Buuren S Multiple imputation of discrete and continuous data by fully conditional specification Stat Methods Med Res 2007 16 3 219 242 17621469 10.1177/0962280206074463

[B86] Vasilaki EI Hosier SG Cox WM The efficacy of motivational interviewing as a brief intervention for excessive drinking: a meta-analytic review Alcohol Alcohol 2006 41 3 328 335 16547122 10.1093/alcalc/agl016

[B87] Woolard R Cherpitel C Kathleen T Brief intervention for emergency department patients with alcohol misuse: implications for current practice Alcohol Treat Q 2011 29 2 146 157 21886943 10.1080/07347324.2011.557978 PMC3163298

[B88] Thush C Wiers R Moerbeek M Ames SL Grenard JL Sussman S Stacy AW Influence of Motivational Interviewing on Explicit and Implicit Alcohol-Related Cognition and Alcohol Use in At-Risk Adolescents Psychol Addict Behav 2009 23 1 146 151 19290699 10.1037/a0013789 PMC3140345

[B89] Gartlehner G Hansen RA Nissman D Lohr KN Carey TS A simple and valid tool distinguished efficacy from effectiveness trials J Clin Epidemiol 2006 59 1040 1046 16980143 10.1016/j.jclinepi.2006.01.011

[B90] Audrey S Holliday J Parry-Langdon N Campbell R Meeting the challenge of implementing process implementation within randomized-controlled trials: The example of ASSIST (A Stop Smoking in Schools Trial) Health Educ Res 2006 21 3 366 377 16740670 10.1093/her/cyl029

[B91] de Vries H Mudde A Kremers S Wetzels J Uiters E Ariza C Vitoria PD Fielder A Holm K Janssen K Lehtuvuori R Candel M The European Smoking Prevention Framework Approach (ESFA): short-term effects Health Educ Res 2003 18 6 649 663 14654499 10.1093/her/cyg033

[B92] Vitale SG van de Mheen H van de Weil A Garretsen HF Substance use among emergency room patients: is self-report preferable to biochemical markers? Addict Behav 2006 31 1661 1669 16446045 10.1016/j.addbeh.2005.12.011

[B93] Harter M Kentgens M Brandes A Bock T Dirmaier J Erzberger M Furstenberg W Hillebrandt B Karow A Von Dem Knesebeck O Konig H-H Lowe B Meyer H-J Romer G Rouhiainen T Scherer M Thomasius R Watzke B Wegscheider K Lambert M Rationale and content of psychenet: The hamburg network for mental health Eur Arch Psychiatry Clin Neurosci 2012 262 Suppl 2 S57 S63 22972562 10.1007/s00406-012-0359-y

[B94] Kaner E Brief alcohol intervention: Time for translational research Addiction 2010 105 6 960 961 20659054 10.1111/j.1360-0443.2009.02848.x

